# The rare entity of cholocystocolonic fistula: a case report

**DOI:** 10.11604/pamj.2021.38.262.27409

**Published:** 2021-03-15

**Authors:** Nikolaos Garmpis, Christos Damaskos, Anna Garmpi, Georgia Sypsa, Dimitrios Mantas

**Affiliations:** 1 Second Department of Propedeutic Surgery, Laiko General Hospital, Medical School, National and Kapodistrian University of Athens, Athens, Greece,; 2 Renal Transplantation Unit, Laiko General Hospital, Athens, Greece,; 3 First Department of Propedeutic Internal Medicine, Laiko General Hospital, Medical School, National and Kapodistrian University of Athens, Athens, Greece,; 4 Department of Radiology, Laiko General Hospital, Athens, Greece

**Keywords:** Cholocystocolonic, fistula, gallbladder, colon, case report

## Abstract

Cholocystocolonic fistulas (CCFs) represent a rare medical entity. Previous inflammatory processes in the abdomen, especially in the gallbladder and surgeries are all related to their appearance. There are not typical findings concerning the clinical image and the therapeutic approach varies between patients. Herein, we present a case of a 46-year-old patient, with a history of perforated duodenal ulcer, suffering from abdominal pain and diarrheas. A computed tomography (CT) demonstrated air inside the biliary system. A laparotomy was conducted to the patient and no complications had occurred. In addition, a review of literature regarding the clinical presentation and the therapeutic options for this disease are discussed in this manuscript in relation to our patient.

## Introduction

The cholocystocolonic fistulas (CCF) consist a rare entity which occurs approximately to 1 to 10.000 cholocystectomies and appears mostly in females during the seventh decade of life [1]. It represents the second most common type of cholecystenteric fistula after the cholocystoduodenal one and its range varies depending different studies from 6.3%-26.5% [2,3]. The clinical presentation is atypical and it includes a variety of symptoms and signs such as abdominal pain, diarrhea, weight loss, jaundice, fever, nausea and other dyspeptic symptoms [1,2]. Thus, its diagnosis often occurs intraoperatively [3]. The atypical clinical presentation, the possible co-morbidities and its rarity render this entity a challenging surgical task. Herein, we report a case of 46-year-old patient presenting with upper abdominal pain, tenderness and a previous history of operated duodenum ulcer perforation.

## Patient and observation

A 46-year-old female patient referred to the emergency department with a 14-day history of abdominal pain in the upper abdomen, including the right upper quadrant and epigastrium. In addition, diarrhea and weight loss were reported from the subject. Her medical history included a perforated duodenal ulcer 3 years earlier, for which she underwent a Billroth's II operation. Postoperatively, she was fit and healthy.

Clinical examination revealed hyperactive bowel sounds during auscultation. Furthermore, tenderness was noticed to both deep and superficial palpation in the epigastrium and right upper quadrant of the abdomen. The rectum was empty when digital rectal examination was performed.

Laboratory findings demonstrated elevated levels of C-reactive protein (CRP): 22mg/L, aspartate aminotransferase (AST): 55IU/L, alanine aminotransferase (ALT): 74IU/L and alkaline phosphatase (ALP): 170mg/L. A left shifted blood cell count with 13.540/mm^3^ was demonstrated. The aerial blood gases (ABGs) showed mild metabolic acidosis.

**Figure 1:**
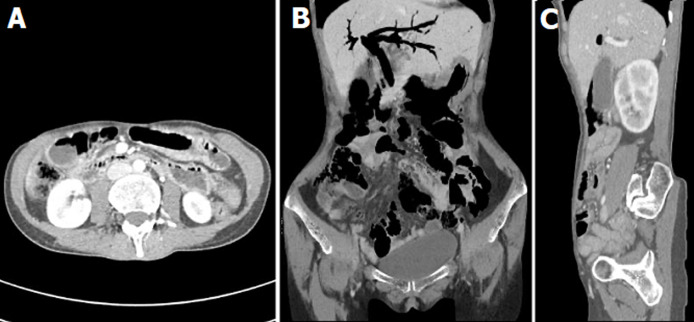
computed tomography findings: A) axial view; B) coronal view; C) sagittal view

The abdominal radiograph did not show any significant abnormality such as pneumobilia or dilated bowels. In the computed tomography (CT), there was no free intrabdominal gas or fluid and a new perforation of an abdominal organ was excluded. The gallbladder was thick, with enhanced wall after contrast administration and contained air inside, which raised the suspicion of a CCF even though no apparent fistulous communication was shown (Figure 1). Initially, due to the previous surgical history of the patient and the possibility of adhesions, conservative treatment was implemented to the patient. As the patient deteriorated and she appeared with symptoms and signs of acute abdomen (or sepsis), a laparotomy was performed. During surgery the existence of a CCF between right colic flexure and gallbladder was obvious and both right colon and gallbladder were surgically removed en-bloc. The postoperative period was uneventful and the patient discharged after 12 days. Seven months postoperatively, the patient remains fit and healthy.

## Discussion

The pathogenesis of the CCF varies. Except for the gall bladder disease and the acute cholecystitis, other conditions correlated with this lesion are previous cholocystectomy, gastric surgery and iatrogenic or traumatic abdominal lesions [1]. In our report, gastric surgery was performed due to a perforated duodenum ulcer. It should be mentioned that there is a reduction of cases reported the last decades since laparoscopy is used more frequently. This is due to the operation of a less invasive surgical method in younger patients [3]. Even though the mean age of patients suffering with this disease is 71-year-old in the Western countries, our patient was only 46 [1]. As far as the clinical presentation is concerned, Hession *et al*. reported that diarrhea appears in 71% of patients [4]. The upper abdominal pain or the presentation of cholangitis have been reported to literature but not in that extent [5]. Our case confirms literature since she was referred to our institution with diarrheas and upper abdominal pain.

The complications of this entity can affect many different systems. Malabsorption leading to osteomalacia, heart failure and megaloblastic anemia, caused by diarrheas are another manifestation of this disease [6]. The most common complication is ileus due to gallstone in the sigmoid colon, which has narrowest diameter [1,7]. Bleeding, pylephlebitis and liver abscesses are other severe complications occurring as a result of a CCF reported in some publications [1,8].

Pneumobilia is the pathognomic radiological sign for the diagnosis of CCF in both abdominal X-ray and CT, even though it is not a frequent finding [9]. In the CT performed in our department, air was detected in the biliary system. Gaillard *et al*. reported that CT-intravenous cholangiography was used for the diagnosis of this disease [10]. Mourri *et al*. diagnosed a CCF through gadoxetic-acid magnetic resonance cholangiography [2]. Abdominal ultrasound (US) rarely offers significant information regarding the CCFs [9]. Okada *et al*. managed to diagnose preoperatively a CCF through endoscopic ultrasound [11]. Except for endoscopic ultrasound (EUS), other endoscopic tools used for detection of CCF are the Endoscopic Retrograde Cholangio Pancreatography (ERCP) and the simple colonoscopy without US [12]. Preoperative diagnosis has also been reported through the use of barium enema [13]. Although all these diagnostic tools have been used for the preoperative diagnosis, none of them has shown great sensitivity [1]. Due to the atypical clinical and imaging signs, the diagnosis of CCF remains a challenge. CCF can be misdiagnosed with malignancies, inflammatory processes, such as diverticulitis or cholecystitis and other etiologies of the complications referred above [1,14].

Therapeutic approaches vary nowadays concerning the treatment of CCF. In non-complicated cases less invasive and aggressive methods are applied in order to minimize the surgical risk. Goldberg *et al*. conducted a therapeutic sphincterotomy [15]. The success of this method is probably due to reduction of bile pressure and spontaneous closure of the fistula. Concerning the surgical treatment, laparoscopy is not a contradiction, but its longer duration and possible complications do not render her superior to laparotomy [12]. Chowbey *et al*. performed laparoscopic surgeries of CCF and reported a 4.8% and 11.1% rate of major and minor complications respectively [1]. Thus, in uncomplicated cases, an open cholecystectomy followed by closure of the fistula is considered as the gold standard treatment approach [16]. In addition, colonic resection can be performed depending on the lesions [17].

Concerning the treatment of the complicated forms, this varies depending on the complication. Enterolithotomy or surgical resection can be conducted on gallstone ileus [5]. It is worthwhile mentioning that surgeons should conduct an extensive exploration of the abdomen of simultaneous lesions such as caecum perforation and other gallstones [18]. The gallstone can also be removed through colonoscopy, a procedure more appropriate for poor condition subjects [19]. A massive hemorrhage, caused by CCF, requires colectomy, cholocystectomy and embolism of the vessel if possible [8]. Our case is worthwhile mentioning because the patient is a young female, without previous cholocystitis and the only risk factor was a previous abdominal surgery. The CT findings guided us to the diagnosis which was confirmed intraoperatively. Our patient underwent laparotomy since it was a complicated case and both gallbladder and right colon were resected.

## Conclusion

The cholocystocolonic fistulas consists a rare disease which should be suspected in many different cases of abdominal pathology, even if its most frequent symptoms, signs and radiological findings are missing. Its preoperative diagnose requires high clinical suspicion and collaboration between different specialties. This is required in order to improve prognosis since its randomly intraoperative discovery is a very demanding surgical challenge with possible dangerous complications for the patient.
